# Effects of zinc supplementation on fatigue and quality of life in patients with colorectal cancer

**DOI:** 10.1590/S1679-45082017AO3830

**Published:** 2017

**Authors:** Sofia Miranda de Figueiredo Ribeiro, Camila Bitu Moreno Braga, Fernanda Maris Peria, Edson Zangiacomi Martinez, José Joaquim Ribeiro da Rocha, Selma Freire Carvalho Cunha

**Affiliations:** 1 Faculdade de Medicina de Ribeirão Preto, Universidade de São Paulo, Ribeirão Preto, SP, Brazil.

**Keywords:** Zinc, Fatigue, Quality of life, Colorectal neoplasms/drug therapy

## Abstract

**Objective:**

To investigate the effects of oral zinc supplementation on fatigue intensity and quality of life of patients during chemotherapy for colorectal cancer.

**Methods:**

A prospective, randomized, double-blinded, placebo-controlled study was conducted with 24 patients on chemotherapy for colorectal adenocarcinoma in a tertiary care public hospital. The study patients received zinc capsules 35mg (Zinc Group, n=10) or placebo (Placebo Group, n=14) orally, twice daily (70mg/day), for 16 weeks, from the immediate postoperative period to the fourth chemotherapy cycle. Approximately 45 days after surgical resection of the tumor, all patients received a chemotherapeutic regimen. Before each of the four cycles of chemotherapy, the Functional Assessment of Chronic Illness Therapy-Fatigue scale was completed. We used a linear mixed model for longitudinal data for statistical analysis.

**Results:**

The scores of quality of life and fatigue questionnaires were similar between the groups during the chemotherapy cycles. The Placebo Group presented worsening of quality of life and increased fatigue between the first and fourth cycles of chemotherapy, but there were no changes in the scores of quality of life or fatigue in the Zinc Group.

**Conclusion:**

Zinc supplementation prevented fatigue and maintained quality of life of patients with colorectal cancer on chemotherapy.

## RESUMO

**Objetivo:**

Investigar os efeitos da suplementação oral de zinco sobre a intensidade da fadiga e a qualidade de vida de pacientes durante a quimioterapia para neoplasia colorretal.

**Métodos:**

Estudo prospectivo, randomizado, controlado e duplo-cego conduzido em um hospital universitário público terciário, com 24 pacientes em regime quimioterápico para adenocarcinoma colorretal. Os pacientes receberam cápsulas de zinco 35mg (Grupo Zinco, n=10) ou placebo (Grupo Placebo, n=14) por via oral, duas vezes ao dia (70mg/dia), durante 16 semanas, desde o período pós-operatório imediato até o quarto ciclo de quimioterapia. Todos os pacientes receberam quimioterapia por aproximadamente 45 dias após a ressecção cirúrgica do tumor. A escala *Functional Assessment of Chronic Illness Therapy-Fatigue* foi preenchida antes de cada um dos quatro ciclos de quimioterapia. Utilizou-se o modelo de regressão linear misto para dados longitudinais para análise estatística.

**Resultados:**

Os escores de qualidade de vida e de fadiga foram semelhantes entre os grupos de estudo durante os ciclos de quimioterapia. O Grupo Placebo apresentou piora da qualidade de vida e da fadiga entre o primeiro e o quarto ciclos de quimioterapia, mas não houve mudança nos escores de qualidade de vida e fadiga no Grupo Zinco.

**Conclusão:**

A suplementação com zinco previne a fadiga e preserva a qualidade de vida de pacientes em quimioterapia para neoplasia colorretal.

## INTRODUCTION

In an advanced disease such as cancer, fatigue can be described as tiredness, weakness, or lack of energy. Fatigue is a distressing symptom, occurring in 39 to 90% of patients on chemotherapy, which may affect their physical, emotional, and/or cognitive function.^1,2^ The pathophysiology of fatigue is not entirely understood and has been related to the release of large amounts of cytokines from the tumor, or to antineoplastic therapy.^3,4^ Fatigue severity increases with consecutive cycles of chemotherapy, may reduce compliance with the planned treatment regimen and have a negative impact on different dimensions of quality of life.^3,5,6^ However, fatigue may be under-recognized by health care professionals, in part due to lack of specific interventional strategies.^2,6^

Zinc plays a major role in various physiological processes, acting as an intracellular signaling molecule, in repair of DNA damage, in cell proliferation, in inhibition of NADPH oxidase, in the structure and stability of some enzymes, in modulating ATP function, and in the maintenance of the immune and anti-inflammatory systems.^7^The activity of several enzymes in energy metabolism requires zinc, and low zinc levels would result in reduced muscle capacity.^8^ An inverse correlation between serum zinc and fatigue has been described in patients after a variety of abdominal surgical interventions.^9^ This result suggests that zinc is implicated, directly or indirectly, in fatigue mechanisms.^9^

## OBJECTIVE

To investigate the effects of oral zinc supplementation on fatigue intensity and quality of life of patients during chemotherapy for colorectal cancer.

## METHODS

**Figure 1:**
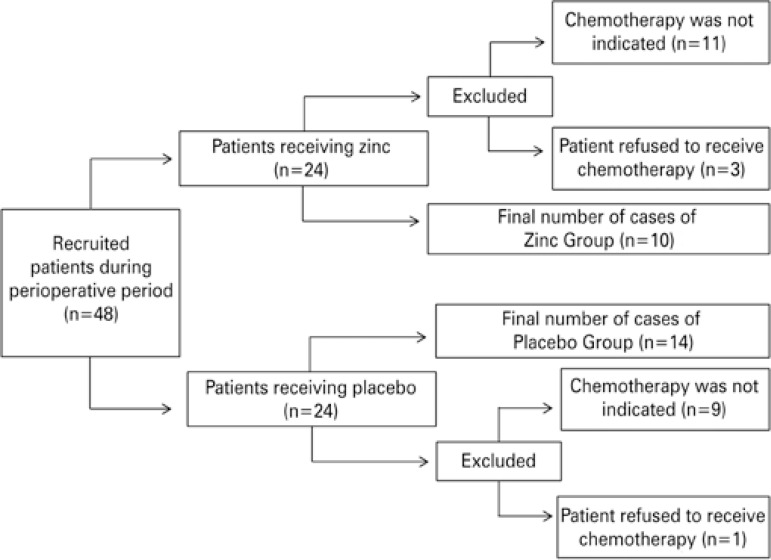
Flow diagram of the study patients

This placebo-controlled, prospective, double-blinded, randomized study was approved by the Research Ethics Committee of a public tertiary hospital (process 14102/2010), and registered with ClinicalTrials.gov, number NCT02106806. All patients provided Informed Consent before participation in the study. Data were collected between May 2011 and December 2012 in oncology care settings. All patients during this period of the study who had undergone surgical resection of the colon or of rectal adenocarcinoma, and were on adjuvant or palliative treatment were eligible for the study. We excluded individuals (i) with liver, kidney, or chronic inflammatory autoimmune diseases; (ii) with active infectious diseases; (iii) who were undergoing therapy with immunosuppressants; (iv) who were on vitamin or mineral supplementation; (v) who had been on chemo- or radiation therapy in the previous 12 months; and (vi) who had been diagnosed as cognitively impaired. A modified Latin-square design was used to allocate 48 individuals to receive capsules containing zinc (Zinc Group) or placebo (Placebo Group) during the study period. Among these volunteers, chemotherapy was not indicated in 19 patients, and 5 individuals refused the chemotherapy. Twenty-four volunteers completed the study (10 in the Zinc Group and 14 in the Placebo Group, according to figure 1).

All participants received chemotherapeutic agents according to the protocol of the Oncology Service: (a) CAPOX (capecitabine + oxaliplatin) schedule; (b) capecitabine; (c) 5-fluorouracil and folinic acid. No participant had a zinc deficiency at baseline. In the perioperative period, no significant differences in demographic characteristics and food intake (by semi-quantitative food frequency questionnaire) were observed between groups (Table 1).

**Table 1:** Demographic and clinical characteristics, and dietary pattern in Zinc and Placebo Groups at baseline

	Zinc Group (n=10)	Placebo Group (n=14)
Age, years	62.5±17.5	63.8±13.3
Gender, male:female	4:6	5:9
Socioeconomic status, (n)		
Lower-middle	8	10
Middle	2	4
Tumor staging, (n)		
II	2	3
III	4	10
IV	4	1
Chemotherapy staging, (n)		
CAPOX (capecitabine + oxaliplatin)	5	11
Capecitabine	3	1
5-fluorouracil + folinic acid	2	2
Dietary intake pattern (Mean±SD)		
Energy, kcal/kg	32.6±9.7	33.6±17.3
Protein, g/kg	1.1±0.5	1.39±0.7
Zinc, mg	8.7±2.3	9.4±4.6

SD: standard deviation.

The study lasted approximately 16 weeks, starting from the beginning of zinc or placebo administration at the early postoperative period. Chemotherapy began approximately 45 days after the start of zinc supplementation or placebo administration. Zinc was administered as zinc sulfate (154mg/capsule), which corresponded to 35mg of elemental zinc. All participants were instructed to take a capsule after breakfast and dinner, which amounted to 70mg of elemental zinc per day. The researchers had no knowledge of the capsule contents until data collection, and statistical analyses were complete.

We determined body composition and laboratory data before the beginning of supplementation (baseline), and at the first and fourth chemotherapy cycles. The body mass index (kg/m^2^) was calculated after measurement of height (m) and body weight (kg) using standardized techniques. The lean mass was obtained by bioelectrical impedance analysis (Biodynamics BIA 450 Analyzer, Biodynamics Corporation, Shoreline, WA, USA). Laboratory data included hemoglobin, mean corpuscular volume, urea, creatinine, albumin, zinc (reference range: 50 to 120µg/dL) and copper (reference range: 70 to 140µg/dL) plasma levels.

To assess fatigue and quality of life, the patients completed the validated, translated into Portuguese version of The Functional Assessment of Chronic Illness Therapy-Fatigue (FACIT-F), a cancer-specific tool,^10^ before each of the four cycles of chemotherapy. The FACIT-F is a 27-item, comprehensive compilation of questions that measure quality of life divided into four domains: physical, social/family, emotional and functional well-being. The tool is a subscale of the general FACIT questionnaire and contains fatigue-related questions. The responses to the items on FACIT-F are each anchored by a five-point Likert scale, running from zero (not at all) to 4 (very much). The scale score was computed by summing the non-missing item scores, multiplying by the total number of items in the scale, and dividing by the number of non-missing items. For overall quality of life, the questionnaire gives total possible scores of zero to 160, in which higher scores indicate better quality of life. Total fatigue score ranges from zero to 52, and high scores represent less fatigue.

We used a linear mixed model for longitudinal data, taking into account random effects. This analysis allowed us to correlate different measurements of each individual at each time, and to compare mean values between groups and times. These comparisons were adjusted by sex, age, and body mass index at baseline. The PROC MIXED procedure of the Statistical Analysis System version 9 (Statistical Analysis System Institute, Cary, NC, USA) software was employed for this purpose. A significance concentration of 5% was set for all analyses.

## RESULTS

In the Zinc Group, plasma zinc increased after supplementation and its concentration was higher than in the Placebo Group before the fourth chemotherapy cycle. There was no difference in body composition and laboratory data in the longitudinal assessment between the study groups, except in the Zinc Group, in which hemoglobin increased before the fourth chemotherapy cycle as compared to baseline (Table 2).

**Table 2:** Body composition and biochemical data in the Zinc and Placebo Groups

	Baseline		Before first chemotherapy cycle		Before fourth chemotherapy cycle	
		
	Group		Group		Group	
		
	Zinc	Placebo	Zinc	Placebo	Zinc	Placebo
Body mass index, kg/m^2^	24.8±5.9	24.9±5.1	23.1±5.1	23.8±5.5	23.9±5.1	24.2±6.5
Lean mass, kg	45.8±10.7	46.6±11.2	45.0±13.0	42.2±8.0	45.6±10.4	42.6±10.2
Hemoglobin, g/dL	10.6±1.2	11.4±1.2	11.3±1.0	11.1±1.6	12.2±1.1*	11.6±1.7
Mean corpuscular volume, fL	84.8±11.9	85.7±6.1	86.4±10.4	84.8±7.9	86.5±22.0	83.7±23.5
Urea, mg/dL	21.9±5.8	27.8±9.4	28.3±7.0	30.9±8.4	27.7±10.4	30.7±11.9
Creatinine, mg/dL	0.9±0.2	0.8±0.2	0.8±0.2	0.7±0.2	0.9±0.2	1.0±0.6
Albumin, g/dL	3.7±0.3	3.7±0.3	3.8±0.6	4.0±0.3	4.1±0.3	3.7±0.4
Zinc, mg/dL	85.2±13.6	75.8±16.2	110.5±24.0*	83.4±17.7	128.9±33.4*†	89.2±19.0
Copper, mg/dL	118.9±23.7	121.9±21.5	120.6±0.6	134.7±34.6	94.2±38.1	119.3±25.1

* Statistical difference in Zinc Group compared to basal time (before supplementation); † statistical difference between Zinc Group *versus* Placebo Group before fourth chemo cycle.

The scores of quality of life and fatigue questionnaires were not different between the groups during the chemotherapy cycles (Table 3). There were no changes in the quality of life or fatigue scores in the Zinc Group during the study. However, longitudinal analysis of the data revealed a worsening in quality of life (126±16 *versus* 116±27; p=0.02) and increase in fatigue (43±6 *versus* 36±13; p=0.02) between the first and fourth cycles of chemotherapy in the Placebo Group.

**Table 3:** Quality of life and fatigue scores during chemotherapy, according to supplementation of zinc (Zinc Group) or placebo (Placebo Group)

Group	Chemotherapy cycle	Quality of life	Fatigue
Zinc Group (n=10)	Before first	121±20	40±9
	Before second	118±17	37±8
	Before third	117±27	36±13
	Before fourth	123±15	40±6

Placebo Group (n=14)	Before first	126±16	43±6
	Before second	123±15	40±7
	Before third	122±21	38±11
	Before fourth	116±27*	36±13*

* Statistical difference between the first and the fourth chemotherapy cycles in the Placebo Group. Statistical test: linear mixed model for longitudinal data.

## DISCUSSION

Patients receiving placebo capsules experienced a worsening of fatigue and quality of life scores during the cycles of chemotherapy. Similar to our findings, prior observations reported that fatigue seemed to increase over consecutive cycles of chemotherapy.^11^ Miller et al.,^3^ showed that the intensity of fatigue is constant during the first three cycles of chemotherapy, and higher in the seventh cycle. The type of antineoplastic drug and the cumulative cytotoxic effects of chemotherapeutic agents may affect fatigue intensity and quality of life.^12-14^

This study showed that oral zinc supplementation 45 days before, and up to 12 weeks after chemotherapy for colorectal cancer, prevents worsening of fatigue symptoms and preserves quality of life. The reduction in the Placebo Group score indicates relevant worsening of fatigue, considering that the minimal clinically important differences for the fatigue scale is 3.0 points.^15^

Zinc deficiency is associated with fatigue intensity in the chronic fatigue syndrome, related to inflammation markers, immune activation and oxidative stress associated with lipid membrane damage.^16-18^On the other hand, zinc sulfate did not change the occurrence of grade 3 fatigue in oral cavity cancer patients who were undergoing radiation therapy.^19^ The quality of life score was similar between zinc-treated and placebo-exposed patients during chemotherapy treatment for different cancers, or radiation therapy for head and neck cancer.^19,20^

Ours was a randomized, double-blinded study, minimizing the chance of bias in sample selection or interpretation of results. We matched the Zinc and Placebo Groups regarding age, sex, socioeconomic status and body composition, since these factors may affect the quality of life of patients in chemotherapy.^21^ At the beginning of the study, all participants had plasma zinc levels within the reference range, excluding the possibility that supplemented zinc was used for recovery of the body’s zinc reserves, rather than producing a supplementary effect. Renal function, anemia, and nutritional status did not change during the study, ruling out the hypothesis that these variables influenced the scores of fatigue and quality of life. The questionnaire used in the present study to assess quality of life and fatigue is based on the sum of subscale scores. Therefore, maintenance of quality of life reported in patients who received zinc supplementation may be, in fact, due to the unchanged severity of fatigue.

This study should be faced as a feasibility trial due to the small sample size. However, its longitudinal design allowed us to identify the effects of zinc supplementation during chemotherapy. We assessed subjects during the initial cycles of chemotherapy and the results obtained could be more evident if the study had been developed during the entire treatment period. Furthermore, the hypothesis that other factors, besides zinc supplementation, may have influenced the results cannot be ruled out. Although participants in adjuvant and palliative treatment were included in our study, no significant differences in quality of life were reported between those treated with adjuvant therapy or palliative intent.^21,22^

## CONCLUSION

Zinc supplementation prevents worsening of fatigue and of quality of life in patients undergoing chemotherapy, after surgery for colorectal cancer. This feasibility trial points to the need to develop further studies with larger sample sizes, and investigation of the biological mechanisms of zinc supplementation on fatigue during chemotherapy.
